# Inverse FASN and LDHA correlation drives metabolic resistance in breast cancer

**DOI:** 10.1186/s12967-024-05517-9

**Published:** 2024-07-24

**Authors:** Chiara Papulino, Ugo Chianese, Ahmad Ali, Gregorio Favale, Concetta Tuccillo, Fortunato Ciardiello, Annabella Di Mauro, Chiara Mignogna, Gerardo Ferrara, Alfredo Budillon, Wouter Leonard Megchelenbrink, Nunzio Del Gaudio, Mariarosaria Conte, Fabrizio Merciai, Pietro Campiglia, Lucia Altucci, Vincenzo Carafa, Eduardo Sommella, Rosaria Benedetti

**Affiliations:** 1 Department of Precision Medicine, University of Campania “Luigi Vanvitelli”, 80138 Naples, Italy; 2 Pathology Unit, Istituto Nazionale Tumori IRCCS Fondazione G. Pascale, 80131 Naples, Italy; 3 Scientific Directorate, Istituto Nazionale Tumori IRCCS Fondazione G. Pascale, 80131 Naples, Italy; 4 Department of Pharmacy (DIFARMA), University of Salerno, 84084 Salerno, Italy; 5 Biogem Institute of Molecular and Genetic Biology, 83031 Ariano Irpino, Italy; 6 Institute of Endocrinology and Oncology “Gaetano Salvatore” (IEOS), 80131 Naples, Italy; 7 Program of Medical Epigenetics, Vanvitelli Hospital, Naples, Italy

**Keywords:** Breast cancer, FASN, LDHA, Metabolism, Tamoxifen resistance

## Abstract

**Background:**

Breast cancer manifests as a heterogeneous pathology marked by complex metabolic reprogramming essential to satisfy its energy demands. Oncogenic signals boost the metabolism, modifying fatty acid synthesis and glucose use from the onset to progression and therapy resistant-forms. However, the exact contribution of metabolic dependencies during tumor evolution remains unclear.

**Methods:**

In this study, we elucidate the connection between FASN and LDHA, pivotal metabolic genes, and their correlation with tumor grade and therapy response using datasets from public repositories. Subsequently, we evaluated the metabolic and proliferative functions upon FASN and LDHA inhibition in breast cancer models. Lastly, we integrated metabolomic and lipidomic analysis to define the contributions of metabolites, lipids, and precursors to the metabolic phenotypes.

**Results:**

Collectively, our findings indicate metabolic shifts during breast cancer progression, unvealling two distinct functional energy phenotypes associated with aggressiveness and therapy response. Specifically, FASN exhibits reduced expression in advance-grade tumors and therapy-resistant forms, whereas LDHA demonstrates higher expression. Additionally, the biological and metabolic impact of blocking the enzymatic activity of FASN and LDHA was correlated with resistant conditions.

**Conclusions:**

These observations emphasize the intrinsic metabolic heterogeneity within breast cancer, thereby highlighting the relevance of metabolic interventions in the field of precision medicine.

**Supplementary Information:**

The online version contains supplementary material available at 10.1186/s12967-024-05517-9.

## Introduction

Breast cancer (BC) stands as the most prevalent malignancy among women, with an estimated annual incidence of 2.3 million new cases and 685,000 deaths globally [1]. BC presents itself as a heterogeneous disease, showcasing various histological and molecular subtypes with distinct risk profile [2–4]. Recent studies have highlighted that cancer heterogeneity is not solely driven by genetic factors but also involves metabolic reprogramming pathways. These pathways are directly involved in energy production and anabolism, facilitating rapid proliferation and other cancer-associated traits such as cytoskeletal dynamics and tumor microenvironment acidity [5–8]. It is noteworthy that not all tumors exhibit identical metabolic dependencies and primary energy substrates. For example, while some tumors may favor glycolysis as their primary energy pathway (known as the Warburg effect), others may predominantly rely on fatty acid oxidation or alternate metabolic pathways to satisfy their energetic demands [9, 10]. Fatty acids (FAs) have diverse roles in cellular activities, serving as energy storage for fueling aerobic respiration through β-oxidation, as well as contributing to cell membrane structure and participating in signal transduction [11]. In BC, FAs have emerged as potential key players in modulating tumor behavior and influencing disease progression [12]. Neoplastic transformation renders BC independent of external sources of FAs, favoring an internal mechanism that catalyzes palmitate synthesis through Fatty Acid Synthase (FASN). FASN, functioning as the rate-limiting enzyme for de novo FA production, enhances the generation of additional energy and facilitates the formation of cell membranes, promoting cancer proliferation and progression [13–16]. To date, only a limited number of studies have endeavored to establish connections between metabolic signatures and mechanisms of therapy resistance in BC [17–20]. In particular, tamoxifen, known for its efficacy and tolerability, is administered for BC chemoprevention and remains the first-line hormone treatment in post-surgery adjuvant therapy [21]. Nevertheless, patients may eventually develop resistance, and the specific mechanisms underlying tamoxifen resistance remain elusive [22]. Evidence suggests a link with altered mitochondrial structure, disassembly of respiratory super complexes, and glycolytic reprogramming as an adaptive metabolism [23, 24]. Acquired tamoxifen resistance in BC cells triggers an anaerobic glycolytic switch, leading to heightened lactate production due to the lactate dehydrogenase A (LDHA) activity coupled to extracellular acidification [25].

In this study, we elucidated the distinct roles of FASN and LDHA as pivotal players in aerobic and anaerobic metabolic pathways, respectively, within BC, correlating their expression profiles with disease progression, aggressiveness, and treatment responsiveness. Our results revealed that FASN predominates under sensitive conditions, crucially contributing to aerobic respiration. However, its activity diminishes in advanced stages and in tamoxifen-resistant conditions. Conversely, the progressive upregulation of LDHA and the prevalence of anaerobic respiration emerged as metabolic signatures associated with the acquisition of tamoxifen resistance. Subsequently, we delineated the functional roles and metabolic adaptability in response to the inhibition of FASN and LDHA using cellular models representative of tamoxifen-resistant BC. Collectively, our findings underscore the metabolic heterogeneity inherent in BC and provide compelling evidence for the implementation of targeted metabolic therapies.

## Materials and methods

### Chemicals

Tamoxifen was purchased from Sigma-Aldrich (Schnelldorf, Germany, #T2859), omeprazole from Selleck Chemicals (Houston, TX, USA, #S1389), and oxamate from Selleck Chemicals (#S6871).

### Cell culture

MCF-7 (HTB-22),MCF-7 TamR (CRL-3435), MDA-MB-231 (HTB-26), and MCF-10A (CRL-10317) cell lines were purchased from ATCC (Milan, Italy). Both cell lines were grown in DMEM (Euroclone, #ECB7501L), supplemented with 10% heat-inactivated fetal bovine serum (Sigma-Aldrich, #F7524), penicillin–streptomycin mix respectively used at 100 U/mL and 100 μg/mL (Euroclone, #ECB3001D), 250 ng/mL amphotericin B (Euroclone, ECM0009D), and 2 mM l-glutamine (Euroclone, ECB3000D). MCF-7 TamR cells were cultured with the addition of 10^–7^ M 4-OH Tamoxifen (Sigma-Aldrich, #68,392–35-8). Cultivation parameters were: 37 °C with 5% CO_2._ Cells analyzed for mycoplasma contamination with EZ-PCR Mycoplasma Test Kit (Biological Industries, #20–700-20).

### Cell lysis and Western blot

Firstly, cell pellets extraction was assesed as described in [26]. Then, cell extract (30 μg) was loaded on 8% and 10% polyacrylamide gels for electophoretic separation and then trasferred on nitrocellulose membranes. Immunoreactive signals were detected with horseradish peroxidase-conjugated secondary antibodies (Bio-Rad, anti-rabbit, #1,705,046, anti-mouse, #1,706,516) and chemiluminescence signals were developed upon ECL (Clarity Western ECL Substrate, 500 ml #1,705,061). Primary antibodies: FASN (#C20G5), LDHA (#C4B5), and GAPDH (#D16H11) from Cell Signaling Technology. Tubulin (#sc-5286) from Santa Cruz Biotechnology. Antibodies were used following the data sheet protocol. ImageJ software (version 1.44) was used to conduct semi-quantitative analysis.

### Cell viability assay

Cell viability in MCF-7 and MCF-7-TamR cell lines was assessed using thiazolyl blue tetrazolium bromide (MTT; Sigma-Aldrich, #57,360–69-7) according to the manufacturer’s instructions. Cells seeded at a density of 4 × 10^3^ cells/well in a 96-well plate. The following day, they were treated with omeprazole and oxamate. Omeprazole was used at final concentrations of 1–5-10–25-50 μM for 24, 48, and 72 h. Oxamate was used at final concentrations of 0.5–1-5–10 mM for 24, 48, and 72 h. In MDA MB-231 and MCF-10A omeprazole and oxamate were tested respectively to 50 μM and 5 mM for 24, 48, and 72 h. Experiments were performed in triplicate and repeated three times. Absorbance values were measured at a wavelength of 570 nm using a Infinite M-plex (Tecan, 30,190,085).

### Cell cycle analysis

2 × 10^5^ cells/mL of MCF-7 and MCF-7 TamR cells were treated with omeprazole 50 μM and oxamate at 5 mM for 24 h and then collected, washed with PBS, and suspended in 500 μL of hypotonic solution (1X PBS, 0.1% sodium citrate, 0.1% NP-40, RNAase A 0.1 mg/ml, and 50 mg/mL PI). The percentage of cell cycle stages were acquired using a BD FACS Celesta Flow Cytometer (BD Biosciences) and analysed for Diva Software. Experiments were performed in triplicate.

### Cell death analysis

MCF-7 and MCF-7 TamR plated at a density of 2 × 10^5^ cells/mL and then treated with omeprazole 50 μM and oxamate at 5 mM for 24 h. After the treatment, cells were collected, washed two times with PBS, and resuspended in PI buffer (0.2 μg/μL PI, PBS 1X). Cell death analysis was assessed by examining the hypodiploid sub-G1 peak and assessing PI incorporation in live cells to determine DNA fragmentation as an early apoptotic event and membrane permeabilization of dead cells as a late apoptotic event, respectively by using FACS Celesta flow cytometer (BD Biosciences). Experiments were performed in triplicate.

### Colony formation assay

MCF-7, MCF-7 TamR, MDA MB-231 cells plated at a density of 1 × 10^3^ cells/well in a 6-well plate. The next day cells were treated with omeprazole and oxamate at 50 μΜ and 5 mM, respectively, for 96 h. Subsequently, cells were cultured for a time over 7 days in a drug-free medium. Then crystal violet was used to stain colonies, and quantification was carried out by dissolving crystal violet with 10% acetic acid. Absorbance values were read at 595 nm (n° replicates = 4) using Infinite M-plex (Tecan, 30,190,085). Statistical significance calculated using the unpaired t- test.

### Trans-well assay

MCF-7 and MCF-7 TamR cells plated at a density of 2 × 10^4^ cells per well in a 24-well plate. The following day, the cells were treated with omeprazole and oxamate at concentrations of 50 μΜ and 5 mM, respectively, for 48 h. After treatment, cells have been collected, counted and seeded (1 × 10^4^ cells) in transwell cell plate as described in [27]. Migrated cells in the lower surface of the membranes were fixed and stained with crystal violet. Then migrated cells have been visualized under microscope at 20× magnification and pictures acquired for rapresentative images.

### Cellular mitochondrial stress and glycolytic rate assay

Metabolic status was investigated on a Seahorse XF96 Analyzer (Agilent Technologies, Santa Clara, CA, USA). Mito Stress Test Kit (Agilent Technologies, #103,015) was used as previously reported in [26] and Glycolytic rate assay kit (Agilent Technologies, #103,344–100) was according to the Agilent protocol. Briefly, 8 × 10^3^ cells were plated 24 h prior to analysis and then treated with omeprazole at 50 μM and 5 mM oxamate for 6 h. The steps for incubation, medium replacement and loading into XF96 Analyzer and injection were performed according to protocol. For Glycolytic rate assay the injection sequence was programmed as reported in the Agilent detailed protocol. Data were analyzed with Wave software version 2.2.0 (Seahorse Bioscience, Agilent Technologies). Experiments were performed in triplicates. Statistical significance was calculated using the unpaired t-test and reported as p-value. Standard deviations reported as error bars.

### Exploration of FASN and LDHA gene expression in normal and BC tissues

Microarray gene expression across normal tissue (NT) and BC downloaded from NCBI (https://www.ncbi.nlm.nih.gov/ by using GEOquery version 2.70.0 [28] as R package in Rstudio version 4.3.3 (http://www.rstudio.com/). Statistical significance calculated using the unpaired t- test. Full list of GSE datasets and samples used in the analysis are provided in Supplementary Table 1. Normalized expression was used to evaluate FASN and LDHA expression in NT and BC condition. Then FASN and LDHA expression was associated to tumor grading. The comprehensive list of GSE datasets and corresponding samples, which report tumor grade and were utilized in the analysis, is provided in Supplementary Table 2.

### Identification of mutations and predictive score

cBioPortal [29] was used to explore SNP mutations, structural variant, amplification, deletion, allele frequency of FASN and LDHA using the search term FASN/LDHA in BC from TCGA PanCancer Atlas Studies. PolyPhen-2 (http://genetics.bwh.harvard.edu/pph2/index.shtml) is a web-accessible resource used for prediction of functional effects of human SNPs. Full list of data are provided in Supplementary Table 3.

### Survival analysis

The association between FASN and LDHA expression, and patients survival was conducted utilizing the Kaplan–Meier Plotter [30]. FASN and LDHA expression was used to divide BC patients in quartiles. The high group comprises samples with gene expression values equal to or greater than the 75th percentile value, while the low group with less than the 75th percentile value. Full list of GSE datasets used as input for the analysis are provided in Supplementary Table 4.

### FASN and LDHA expression in sensitive and insensitive condition.

RNA-seq and microarray transcriptomic analysis downloaded from NCBI (https://www.ncbi.nlm.nih.gov/) by using GEOquery [28]. GSE111151, GSE131276, GSE125738, GSE106681, GSE115737 datasets were used to evalute normalized expression for FASN and LDHA, and their correlation upon sensitive and insensitive condition to tamoxifen.

### FASN and LDHA in positive and negative outcome of BC patients

Expression of FASN, LDHA, and their interactors in partial remission (PR) and progressive disease (PD) condition, respectively as positive and negative outcome was provided from microarray dataset GSE82172. Differential expression analysis was estimated comparing PD against PR group and showed as foldchange. Data have been analyzed with GEOquery [28] and limma version 3.42.2 10 [31] as R package. For RNA-seq data, DESeq2 [32] using R was used to compare gene expression across sample subgroups, we employed the DESeq2 package, utilizing a negative binomial model for detecting differentially expressed genes (DEG) from count data.

### Analysis of protein–protein interactions and functional protein partners

STRING (https://string-db.org/) was used for protein–protein interaction analysis and to determine the top20 functional protein partners of FASN and LDHA, ranked for score. Full list of FASN-LDHA interactors are provided in Supplementary Table 5.

### Correlation analysis

Pearson correlation was evaluated using GraphPad Prism version 8.3.0.

### Patients’ enrolment, ethics approval and consent to participate

The study encompassed a cohort of 104 patients diagnosed with BC. Formalin-fixed, paraffin-embedded tissue blocks from surgical cases between 2014 and 2022 were chosen from Pathology Laboratory. The cohort includes 52 luminal cases, 10 of them exhibiting HER2 overexpression, and 42 categorized as triple negative. The patients’ characteristics are reported in Supplementary Table 6. The study received approval by the Ethics Committee of University of Campania “Luigi Vanvitelli” (protocol 384/2019) and of IRCCS Pascale (Protocol 57/2021) before the beginning of the study, in accordance with the code of Ethics of the Declaration of Helsinki.

### Tissue array

Haematoxylin and Eosin (H-E) stained slides were prepared from original paraffin blocks and analysed for BC diagnosis. The Tissue Microarray (TMA) was designed by selecting the most representative areas, as determined by the pathologist. For each block, two tumor cores and one healthy tissue core, each measuring 1.00 mm, were precisely positioned in a donor block using the GALILEO TMA CK2500 semi-automated instrument.

### Immunohistochemistry

The tissue array was prepared using the following steps: deparaffinization in xylene, hydration in graded alcohol, and antigen retrieval in retrieval buffer containing EDTA (pH 8.0) at 100 °C. This was followed by a 15-min incubation with 5% H_2_O_2_. A 5-min protein block was then performed using 5% BSA in 1 × PBS. Subsequently, the slides were incubated for 1 h with rabbit monoclonal anti-human antibody FASN (dilution 1:200) and mouse monoclonal anti-human antibody LDHA (dilution 1:650). The corresponding horseradish peroxidase (HRP)-conjugated secondary antibodies were then incubated for 40 min at room temperature, followed by visualization with diaminobenzidine (DAB) reagent. Tissue array slides were subsequently stained with haematoxylin and coverslipped for microscopic evaluation. To validate the array results, staining was replicated on a cohort of 38 whole-section blocks. The density of FASN and LDHA staining was assessed both by an experienced pathologist and through digital image analysis. Immunostaining values were reported as the percentage of positive cells. The percentage of positive cancer cells in each sample was determined by counting the number of positive cells over the total number of cancer cells in ten non-overlapping fields at 40 × magnification. Slides were scanned and photographed using the Aperio Scanscope CS (Aperio Technologies^®^, USA). Statistical significance calculated using the unpaired t- test.

### Metabolites and lipids extraction

For metabolites and lipids extraction 1 × 10^6^ cells were plated in 6-well plates,and then treated for 24 h with omeprazole 50 μM and oxamate 5 mM for 24 h. Cells were washed twice with 1 mL of ice-cold PBS per well. Then, 500 μL of cold cell extraction solution (80% methanol/20% water, v/v) was added to each well. The cells were scraped off using a cell scraper, snap-frozen in pre-chilled tubes, and stored in a − 80 °C freezer. For metabolome and lipidome extraction, 225 µL of ice cold MeOH containing a mixture of deuterated standards were added to cell pellets (1 × 10^6^ cells) and incubated at − 30 °C for 1 min and, subsequently, put in a sonic bath for 10 min. After adding 750 µL of ice-cold MTBE, samples were re-incubated in a Thermomixer (Eppendorf) for 1 h × 4 °C × 550 rpm. Afterward, 188 µL of H_2_O was added to the samples. Following centrifugation at 4 °C and 14,680 rpm, the lower and upper phases were separately collected and dried using a SpeedVac (Savant, Thermo Fisher Scientific).

### Metabolomics and lipidomics analysis

Metabolomics analyses were conducted using a Vanquish Flex UHPLC coupled online to a Exploris 120 hybrid quadrupole Orbitrap mass spectrometer (Thermo Fisher Scientific) equipped with a heated electrospray ionization probe (HESI II). Ultimate RS 3000 UHPLC (Thermo Fisher Scientific), coupled online to a TimsTOF Pro Quadrupole Time of Flight (Q-TOF) (Bruker Daltonics) equipped with an Apollo II electrospray ionization (ESI) probe was to perform lipidome analysis. Lipid separation was performed as previously described in [33]. MS acquisition was performed in DDA PASEF mode. Metabolome separation was carried out with a Sequant ZIC-HILIC (100 × 2.1 mm; 3 μm) protected with a precolumn (5 × 21 mm; 3 μm) (Supelco). MS acquisition was performed in DDA mode. Detailed LC and MS conditions and pre-processing steps (alignment, filtering, normalization and annotation) are reported in [33], [34]. Quality control check was assessed injecting in randomically order the samples and blank to exclude background signal. Following data pre-processing, statistical analysis was performed by Metaboanalyst 5.0.

### Enrichment analysis

Metabolite enrichment and network pathway analysis was performed to directly investigate significantly enriched group with functionally related metabolites using MetaboAnalystR package [35]. Compound lists used as input file returned statistically enrichments for each matching dataset.

### Quantification of lipid accumulation with Red Oil staining

MCF-7 and MCF-7 TamR cells were plated at a density of 5 × 10^4^ cells/well in a 12-well plate. The following day, the cells were treated with omeprazole 50 µM for 24 h. Then, 4% formaldehyde in PBS was added for 1 h followed by wash step with distilled water and cells fixed with 60% isopropyl alcohol for 5 min. Further, the cells were stained for 15 min at room temperature with a solution of 0.3% Oil Red O in 60% isopropyl alcohol, followed by wash step with distilled water. Lipid amount was defined dissolving samples in isopropanol for 15 min measuring absorbance with Infinite M-plex (Tecan, 30,190,085) at 540 nm.

### Lactate assay measurement

MCF-7 and MCF-7 TamR cells were plated at a density of 4 × 10^3^ cells per well in a 96-well plate. The following day, the cells were treated with oxamate 5 mM for 48 h. Total and intracellular lactate levels, with and without LDHA inhibition, were measured with Pierce LDH Cytotoxicity Assay Kit (ThermoFisher Scientific, 88,953) following the manufacturer's instructions. Absorbance values were recorded in the range of 490–680 nm using a Infinite M-plex (Tecan, 30,190,085). The extracellular lactate level was determined by subtracting the intracellular lactate absorbance from the total lactate absorbance.

## Results

### FASN and LDHA are overexpressed in BC

**Fig. 1:**
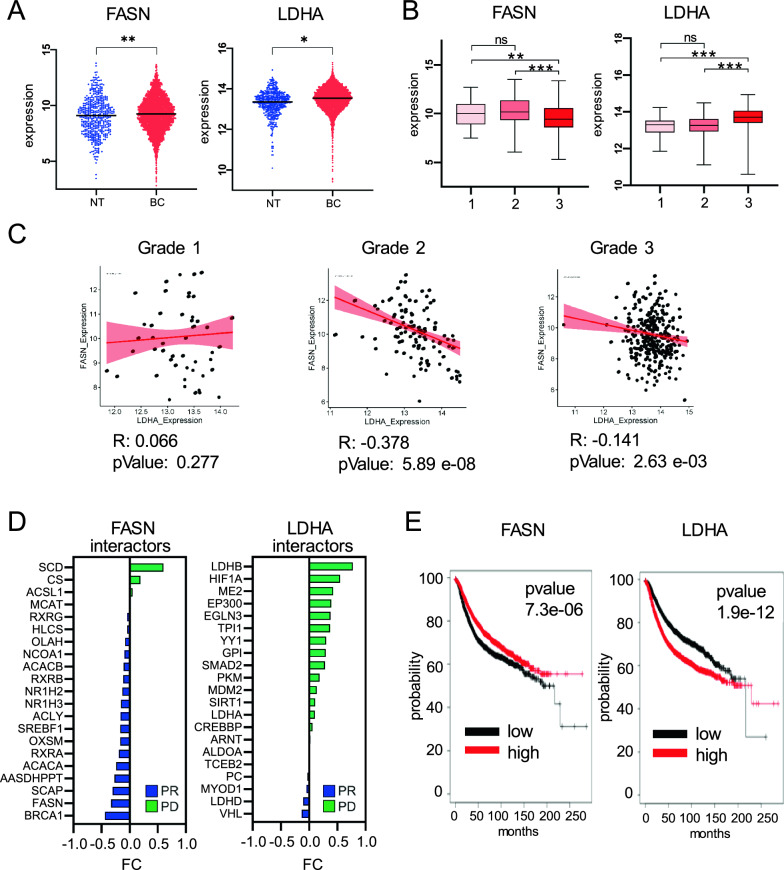
FASN and LDHA expression inBC evolution andsurvival. **A** Nomalized expression of *FASN* and *LDHA* in NT and BC tissues. Statistical significance showed as *p value < 0.05; **p value < 0.01. **B** Nomalized expression of *FASN* and *LDHA* across tumor grade in BC tissue. Statistical significance showed as **p value < 0.01; ***p value < 0.001; ns, not significant. **C** Pearson correlation for FASN and LDHA expression across tumor grade in BC. Statistical significance reported as pvalue. **D** Differential expression analysis for FASN and LDHA with relative interactors calculated as foldchange between PD and PR groups. **E** Kaplan–Meier curve showing survival probability in BC patients based on *FASN* and *LDHA* expression (black = low; red = high)

Microarray data were used to investigate FASN and LDHA expression levels in normal tissue (NT) and BC (NT = 475, BC = 5574) (Supplementary Table 1). FASN and LDHA expression was significantly higher in BC compared to NT (Fig. 1A) and this data was confirmed in our cohort of BC samples (Supplementary Fig. 1A, B). Mutational profiles were also analyzed, returning single nucleotide polymorphism (SNP) as the most prevalent type of variation in both FASN and LDHA genes (Supplementary Fig. 1C), characterized by low allele frequency (Supplementary Fig. 1D). Overall, missense mutations predominated as the most common mutations, with FASN exhibiting a higher mutation rate compared to LDHA (Supplementary Fig. 1E). Notably, in 36.61% of cases, missense mutations in FASN were predicted to exert potential detrimental effects on the functional properties of the protein (Supplementary Fig. 1F and Supplementary Table 3). Conversely, missense mutations in LDHA were less frequent and were predicted not to have a significant functional impact. Collectively, these findings indicate that FASN and LDHA are overexpressed in BC, with a relatively low mutation profile primarily characterized by missense mutations.

### FASN and LDHA expression define metabolic BC subtypes with distinct aggressiveness and patient survival

Transcriptomic analysis was conducted on 725 BC samples, categorized by tumor grade, to explore the expression patterns of FASN and LDHA across the progression of the disease (Supplementary Table 2). FASN expression exhibited downregulation in grade 3 tumors compared to both grade 1 (p-value = 0.01) and grade 2 (p-value = 0.001) tumors, whereas LDHA expression demonstrated an increase in grade 3 tumors relative to grade 1 (p-value = 0.001) and grade 2 (p-value = 0.001) tumors (Fig. 1B). FASN and LDHA expression were uncorrelated at grade 1 (r = 0.07, p-value = 0.28), but negatively correlated in grade 2 (r = − 0.38, p-value = 5.9 e−08) and grade 3 (r = − 0.14, p-value = 2.6 e−03) tumors (Fig. 1C). FASN and LDHA expression patterns was also examined in tamoxifen-resistant conditions in BC patients experiencing partial remission (PR) and disease progression (PD) (Supplementary Fig. 1G). Top 20 functional protein interactors for FASN and LDHA ranked by score (Supplementary Table 5) have been used to define metabolic patterns in these two clinical outcomes. Results showed that FASN and its interactors were enriched in PR patients whereas LDHA and its interactors were enriched in PD patients (Fig. 1D). A negative correlation between FASN and LDHA expression was confirmed in independent RNA-seq and microarray datasets related to tamoxifen sensitivity in BC. Specifically, FASN expression was found to be elevated in tamoxifen-sensitive BC conditions, while LDHA expression was upregulated in tamoxifen-resistant conditions (Supplementary Fig. 2). Within our study cohort, we observed a negative correlation between FASN and LDHA expression in the Luminal B subtype of BC (Supplementary Fig. 3A), although this association did not reach statistical significance. However, Luminal B tumors with higher expression levels of FASN and LDHA tended to exhibit features indicative of an invasive phenotype (IVP) and markers of necrosis (Supplementary Figs. 3B). Overall survival was investigated in our cohort as well, data showed that low FASN expression and high LDHA expression were associated to poor survival rates (Fig. 1E). Similarly, our cohort analysis demonstrated a notable correlation between elevated LDHA expression and adverse prognosis (Supplementary Fig. 3C). These findings unveil metabolic dependencies driven by the expression of FASN and LDHA and their interactors, which are inversely correlated with tumor progression and therapy response. Specifically, the glycolytic phenotype linked to anaerobic respiration is correlated with worse clinical outcomes and patient survival in BC.

### Tamoxifen-sensitive and -resistant BC display differential vulnerability to LDHA and FASN inhibition

**Fig. 2:**
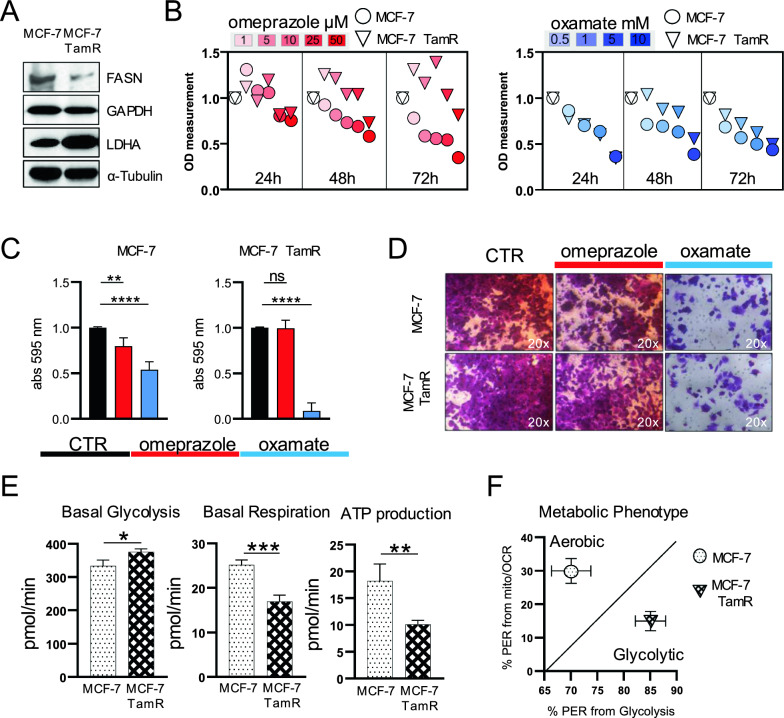
In vitro evaluation on proliferative and metabolic ability in tamoxifen-sensitive and resistant models. **A** Western blot analysis of FASN and LDHA expression in BC tamoxifen-sensitive (MCF-7) and -resistant (MCF-7 TamR) cells. **B** Viability assay in MCF-7 (circle) and MCF-7 TamR (triangle) cells upon FASN inhibitor omeprazole (scale of red) and LDHA inhibitor oxamate (scale of blue). **C** Colony formation assay in MCF-7 and MCF-7 TamR after omeprazole (red) and oxamate treatment (blue). Values are mean ± standard deviation (SD) of biological replicates. Statistical significance showed as **p value < 0.01; ns, not significant. **D** Representative images of Trans-well assay by light microscopy (20 × magnification) in MCF-7 and MCF-7 TamR after omeprazole and oxamate treatment. **E** Basal Glycolysis, Basal Respiration and ATP production in tamoxifen-sensitive and tamoxifen-resistant condition. Values are mean ± standard deviation (SD) of biological replicates. Statistical significance showed as *p value < 0.05, **p value < 0.01, ***p value < 0.001. **F** Metabolic phenotypes defined using proton efflux rate (PER) from Glycolysis and Respiration in MCF-7 (circle) and MCF-7 TamR (triangle)

MCF-7 and MCF-7 TamR BC cell lines, as tamoxifen sensitivite and resistance in vitro models, were used to explore the correlation between FASN/LDHA expression and tumor aggressiveness. MCF-7 TamR cells exhibited low FASN and high LDHA expression compared to MCF-7 cells (Fig. 2A), confirming difference in BC aggressiveness. FASN and LDHA inhibitors, omeprazole and oxamate respectively, were then tested for their potential anticancer effects on BC models. The FASN inhibition resulted in a more pronounced reduction in proliferation in MCF-7 cells compared to MCF-7 TamR cells, whereas inhibition of LDHA exhibited a similar antiproliferative effect in both cell lines (Fig. 2B). Interestingly, the colony-forming ability was notably impacted by FASN inhibition in MCF-7 cells, without altering MCF-7 TamR cells. Conversely, LDHA inhibition led to significant downregulation in both models, albeit more profoundly in MCF-7 TamR cells (Fig. 2C). Omeprazole and oxamate were tested in the MDA MB-231 triple-negative breast cancer (TNBC) cell line model. The results demonstrated significant reductions in cell proliferation induced by oxamate. Furthermore, the LDHA inhibitor exhibited a more pronounced impact on colony formation compared to the FASN inhibitor (see Supplementary Fig. 4A, B), emphasizing the critical role of metabolism even in hormone receptor-deficient contexts and the correlation aggressiveness- glycolytic phenotype in BC. Interestingly, in a non-tumor breast cell system (MCF-10A model), the anti-proliferative effects observed over short and long periods were substantially weaker at equivalent concentrations used in MCF-7 TamR, MCF-7 and MDA MB-231 cells (see Supplementary Fig. 4C). This observation substantiates the heightened susceptibility of breast cancer cells to metabolic inhibitors and underscores the pivotal involvement of metabolic pathways in tumorigenesis. Furthermore, this also implies that in TNBC, characterized by inherent resistance to tamoxifen treatment owing to ERα negativity, metabolic interference could be considered a viable therapeutic strategy. Next, to further corroborate the data in both MCF-7 TamR and MCF-7 models, we proceeded with direct measurements of both lactate and cellular lipids. We assessed lipid accumulation following FASN inhibition, observing a more pronounced increase in lipid vesicles in MCF-7 TamR cells compared to MCF-7 cells (Supplementary Fig. 4D). Lactate assays further confirmed higher extracellular levels in MCF-7 TamR cells, and inhibition of LDHA significantly reduced lactate levels in both cell systems (Supplementary Fig. 4E, F).

This finding supports the hypothesis of decreased lipolysis and a shift towards a glycolytic phenotype in tamoxifen-resistant conditions. Furthermore, FASN inhibition led to a more evident reduction in MCF-7 migration ability, while LDHA inhibition mediated a strong impairment in both systems (Fig. 2D). Cell cycle analysis showed that FASN and LDHA inhibition resulted in a reduction in S and G2M phase in both BC systems, with an increase in G0/G1 (Supplementary Fig. 5A) and an higher percentage of cell death following the LDHA inhibition (Supplementary Fig. 5B). Susequently we have experimentally assessed the direct impacts of these inhibitors on cellular metabolism, showing that basal glycolysis levels exhibited a significant increase in MCF-7 TamR compared to MCF-7 cells. Conversely, MCF-7 cells demonstrated higher cellular respiration, correlating with an elevated yield in ATP production (Fig. 2E). The integrated data uncovered two distinct metabolic dependencies: MCF-7 cells demonstrated a predominantly aerobic phenotype, while MCF-7 TamR cells exhibited a more glycolytic profile (Fig. 2F). These findings highlight that tamoxifen-sensitive and -resistant BC show a different vulnerability to metabolic treatments, and that this heterogeneity is associated to metabolic pathway dependency.

### FASN and LDHA inhibition highlights metabolic flexibility in therapy-resistant vs. therapy-sensitive BC

**Fig. 3:**
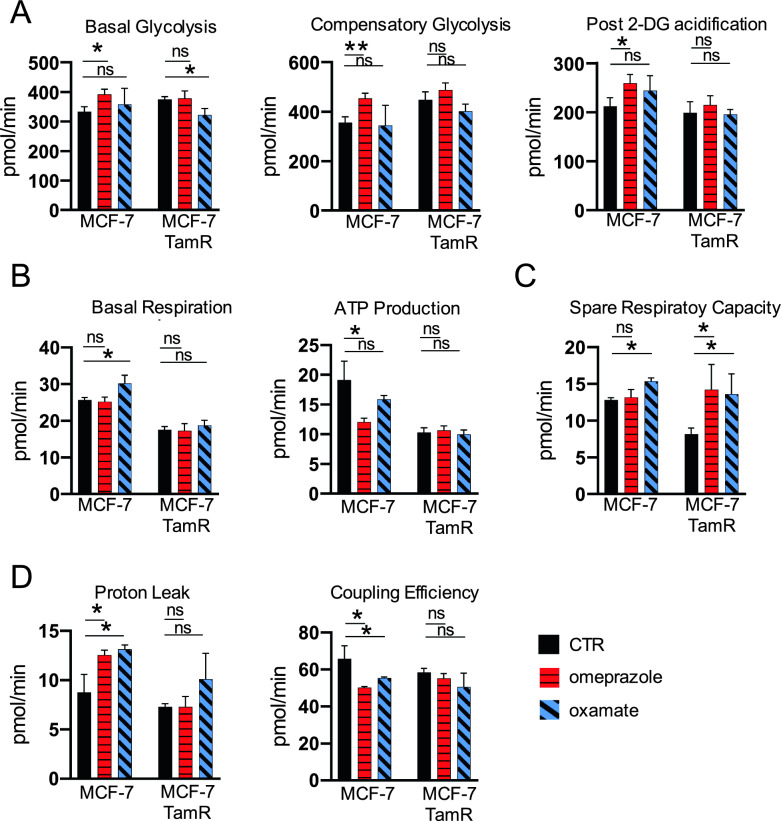
Metabolic response and adaptability upon FASN and LDHA inhibition in sensitive and resistant condition. **A-D** Basal Glycolysis, Proton Efflux Rate, Compensatory Glycolysis, Post 2-DG acidification, Basal Respiration, ATP production, Proton Leak, Coupling Efficiency, Spare Respiration Capacity, mitoOCR/glycoPER, in MCF-7 and MCF-7 TamR after omeprazole (red) and oxamate (blue) treatment. Values are mean ± standard deviation (SD) of biological replicates. Statistical significance showed as *p value < 0.05,**p value < 0.01, ***pvalue < 0.001, ns not significant.

The metabolic adaptability, and specifically the anaerobic and aerobic respiration, were monitored following the inhibition of FASN and LDHA in both BC systems (Supplementary Fig. 5C, D). As showed in Fig. 3A, basal glycolysis increased in MCF-7 cells following FASN inhibition, accompanied by a related increase in compensatory glycolysis with post 2-DG acidification. However, no variations were observed in MCF-7 TamR cells. Conversely, LDHA inhibition did not affect MCF-7 cells, while in MCF-7 TamR cells, basal glycolysis was significantly reduced, with a trend towards compensatory glycolysis with post 2-DG acidification, although not statistically significant. Data pertaining to aerobic respiration revealed notable differences as well. Basal respiration increased in MCF-7 cells upon LDHA inhibition, whereas FASN inhibition was associated with reduced ATP production, with no effect observed in MCF-7 TamR cells (Fig. 3B). Spare respiratory capacity, indicative of metabolic flexibility, exhibited a significant increase in MCF-7 TamR cells following both FASN and LDHA inhibition, while in MCF-7 cells, it was significant only upon LDHA inhibition (Fig. 3C). Substantial differences were noted in proton leak and coupling efficiency, as both treatments led to an increase in proton leak and a reduction in coupling efficiency (Fig. 3D). Taken together, these data highlight metabolic heterogeneity in BC, showing difference in terms of response and metabolic flexibility upon the transition between therapy-resistant and therapy-sensitive neoplasms.

### Fatty acid utilization dominates glucose metabolism in tamoxifen-resistant BC

**Fig. 4:**
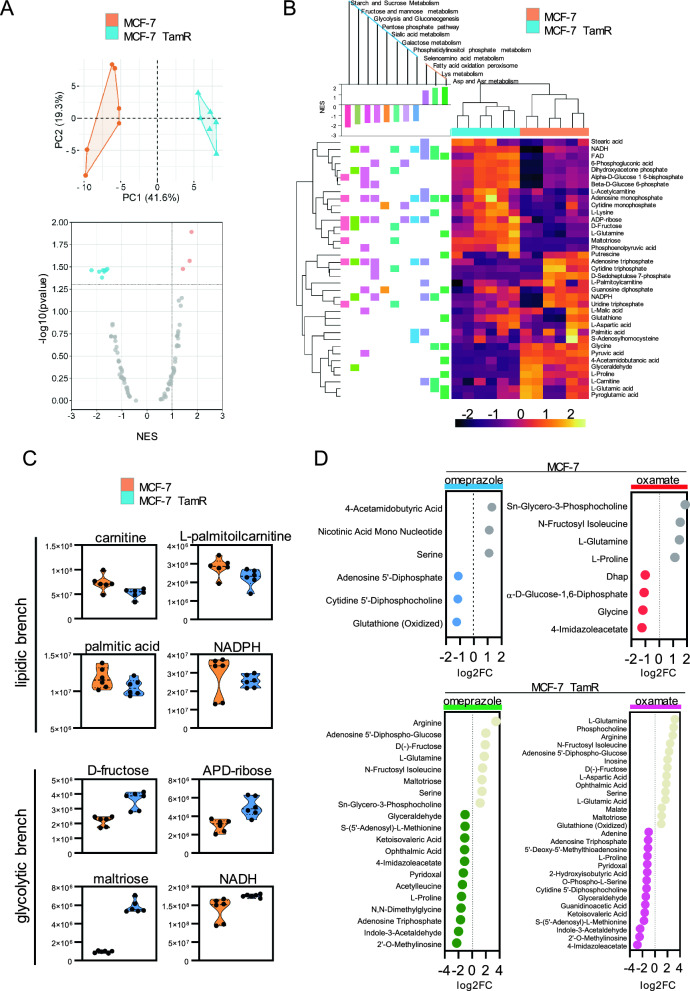
Metabolomic profiling in tamoxifen resistance. **A** Principal Component Analysis of metabolome analysis in MCF-7 and MCF-7 TamR at basal level (upper side). Volcano plot evidencing the main metabolic pathways enriched in the two systems (lowerside). **B** Gene Set Enrichment Analysis for statistically significant differentially expressed metabolites in MCF-7 and MCF-7 TamR cells reporting normalized enriched score (NES). **C** Metabolite expression involved in lipid and glycolytic activity in MCF-7 and MCF-7 TamR at basal level. **D** Significant metabolites from comparison between omeprazole (blue), and oxamate (red) treatment in MCF-7 and MCF-7 TamR. Data reported as log2Fold Change.

Following the identification of two distinct metabolic profiles, metabolites were directly measured in MCF-7 and MCF-7 TamR at basal level and following the inhibition of LDHA and FASN (Supplementary Fig. 6A, B). Functional analysis revealed the critical involvement of glucose, fatty acids, and amino acids in BC metabolism (Supplementary Fig. 6C), delineating a network wherein these molecules acted as connecting nodes within the most relevant energy circuits (Supplementary Fig. 6D). At the basal level, principal component analysis (PCA) showed good variance in the two models (Fig. 4A) and enrichment analysis defined statistically differences in pathways (Fig. 4B). In MCF-7 cells, FA oxidation and peroxisome function emerged as the primary metabolic pathway. In contrast, MCF-7 TamR cells exhibit a significant metabolic shift towards glycolysis and gluconeogenesis. The enrichment analysis revealed significant metabolites involved in FAs metabolism, including L-carnitine, L-palmitoyl-carnitine, and palmitic acid, which were more abundant in MCF-7 cells. Conversely, MCF-7 TamR cells predominantly expressed metabolites such as D-fructose, ADP-ribose, and maltotriose (Fig. 4C). Furthermore, NADPH, primarily utilized in anabolic processes, particularly in lipid biosynthesis reactions, was higher in the sensitive model, whereas NADH, a product of glycolysis, was more abundant in the resistant condition. Further distinctions were related to amino acid pathways, enriched solely in MCF-7 cells, while the pentose phosphate pathway (PPP) and glycolytic processes were predominantly observed in MCF-7 TamR cells. Metabolic content was investigated upon target metabolic as well. FASN inhibition reduced amino acid metabolism in both sensitive and resistant condition, simultaneously activating glycolytic-related processes such as PPP, while biological differences involved β-oxidation of unsaturated FAs increased in MCF-7 cells while MCF-7 TamR had pyruvate metabolism increased (Supplementary Fig. 7A). In MCF-7 cells, LDHA inhibition reduced amino acid metabolism, modulating TCA cycle, glycidic processes and PPP, whereas in MCF-7 TamR cells, the same treatment drastically abrogated amino acid metabolism and reprograms FA oxidation peroxisome (Supplementary Fig. 7B). Among the altered metabolites, serine levels decreased with FASN inhibition in both models, whereas with LDHA inhibition, serine reduction was observed only in MCF-7 TamR (Fig. 4D). This observation indicates a metabolic phenotype geared towards using fatty acids as the primary energy source prioritizing glucose metabolism in tamoxifen resistance condition.

### Lipids profile changes in therapy-resistant vs. therapy-sensitive BC

**Fig. 5:**
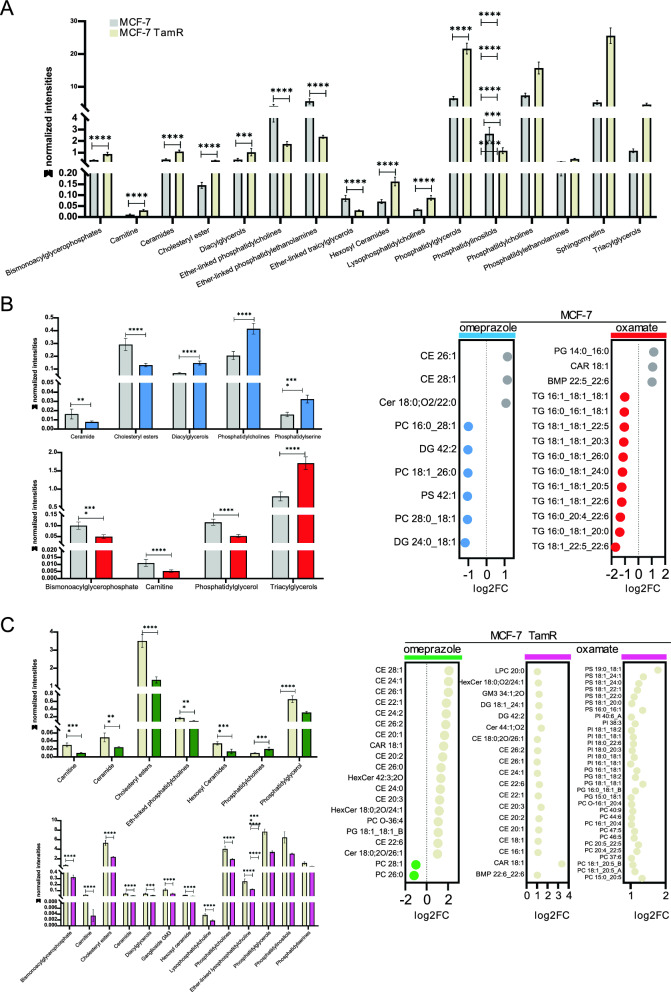
Differences in lipid content resistance-associated. **A** Total abundance for lipid species in MCF-7 (grey) and MCF-7 TamR (yellow). Statistical significance was calculated using the unpaired t- test ****p-value < 0.0001. Data reported as normalized intensities. Values are mean ± standard deviation (SD) of biological replicates. **B** Amount of lipid species altered with omeprazole (blue), and oxamate (red) treatment in MCF-7. **C** Amount of lipid species altered with omeprazole (blue), and oxamate (red)treatment in MCF-7 TamR

Lipidomic amount content was measured at basal level and upon enzymatic inhibition as well. Analysis showed a differential expression of lipid species in the tamoxifen sensitive and resistant cell lines such as ceramides (Cer), diacylglycerols (DG), tryacylglycerols (TG), phosphatidylglycerol (PDG) most abundant in MCF-7 TamR whereas ether-linked phosphatidyl-cholines (PC),ethanolamines (PE) and –inositols (PI) were enriched in MCF-7 (Fig. 5A). Particularly in MCF-7, FASN inhibition significantly reduced cholesteryl esters, increasing DG, phosphatidylserines (PS) and phosphatidylcholines (PC) while LDHA inhibition increased lipid production, specifically triacylglycerols (TG) family, partially affecting prostaglandins (PG), acylcarnitines (CAR), and bismonoacylglycerophosphate (BMP) (Fig. 5B). In MCF-7 TamR cells, FASN inhibition widely downregulated lipid groups such Cer, cholesteryl esters (CE), and hexosylceramides (HexCer), while LDHA inhibition amplified the effect as well as leading to a significant reduction in PC, PG, phosphatidylinositol (PI) and DG (Fig. 5C). All together, these data indicate that aerobic and anaerobic metabolisms in treatment-susceptible BCs are differentially influenced by amino acid utilization, showcasing variability in their capacity to adapt in response to therapy.

## Discussion

BC is a complex multifactorial disease with a severe impact on global public health and society. It now ranks first worldwide in incidence rate, accounting for 32% of all new cancer cases [36]. Over the years, the identification of various therapeutic targets, primarily hormone receptors, has significantly influenced the selection of drug treatments for BC. Estrogen receptor-positive BC, which constitutes approximately 70% of all cases, is commonly treated with tamoxifen, one of the most widely used adjuvant hormonal therapies [37]. However, at least 25% of patients initially responder to tamoxifen treatment develop acquired anti-estrogen resistance. In fact, approximately 50% of patients with metastatic BC, which is the leading cause of mortality, exhibit acquired resistance to tamoxifen. Improving patient stratification and identifying new strategies to overcome drug resistance in BC is thus an urgent need [38, 39]. Since the discovery of metabolic alterations in cancer, the Warburg effect, metabolism has been recognized as a hallmark of cancer. The reprogramming of cellular metabolism, whether directly or indirectly driven by oncogenic mutations, is essential for tumorigenesis. The potential use of metabolic modulators in combination cancer therapies is still in its early stages. However, a better understanding of metabolic dependencies in specific tumor tissues could elucidate and define the metabolic aspects that most limit tumor growth, thereby encouraging preclinical validation and clinical studies [40, 41].

In the study, we evaluate the biological effect upon inhibition of two metabolic enzymes, FASN and LDHA, involved respectively in aerobic and anaerobic respiration for a better comprehension about metabolic dependency and vulnerabilities in hormone therapy resistance BC. We showed that tumorigenic transformation regulates the expression levels of FASN and LDHA with different evolution after onset through progression disease confirming this observation in several independent studies. Specifically, FASN with main functional protein interactors resulted as enriched in patients with partial remission and reduced in advanced tumors whereas LDHA and its interactors were overexpressed in patients with progressive disease. This data suggests that the evolution of metabolism towards anaerobic respiration is associated with BC progression and aggressiveness. Indeed, survival rates indicate that low levels of FASN and high levels of LDHA are linked to poor prognosis in BC patients. These findings underscore the concept of consecutive metabolic reprogramming as an acquired trait over time in response to drug resistance, highlighting the correlation between survival and metabolic evolution. Experimentally, we validated the divergent metabolic phenotypes in sensitivite and tamoxifen-resistance BC cellular models. Our data confirmed distinct expression levels for FASN and LDHA, and different response to target inhibition. If FASN inhibition affected the proliferation and colony-forming ability of the tamoxifen-sensitive system, it did not compromise the resistant model. At the energetic level, sensitivity model exhibited a more aerobic metabolic profile, instead more glycolytic upon tamoxifen resistance highlighting a distinct resistance-related metabolic dependency. Basal respiration and glycolysis after target metabolic treatment suggested a different metabolic adaptability as well. In the sensitive model, the blockage of FASN triggered a glycolytic rebound whereas the blockage of LDHA increased the aerobic metabolism. Oppositely, tamoxifen-resistant model suffered uniquely upon LDHA inhibition resulting in a drop of glycolysis. Furthermore, coupling efficiency and proton leak, as indicators of mitochondrial performance, were altered uniquely in the sensitive model. Additionally, tamoxifen-resistant model showed a better adaptability to metabolic intereferences. This suggests greater mitochondrial vulnerability in the sensitive condition and adaptability related to resistance highlighting important metabolic differences and dependencies that contribute to the heterogeneity of BC. Furthermore, we also investigated the relationship between metabolites and lipids associated to metabolic phenotype looking for a direct connection with metabolome and lipidome. Serine is a crucial amino acid working at several nodes in biological processes, such as the synthesis of lipid classes including sphingolipids, which are now recognized as key players in regulating both cell death and cell survival [42, 43]. Ceramides have also been linked with cell survival and proliferation in a several cancers, including BC [44]. In our dataset, serine was downregulated upon FASN inhibition in the two resistant conditions, but only in MCF-7 TamR cells upon LDHA inhibition. As a precursor, serine reflected ceramide production at lipid level. In the sensitive system, the blockade of LDHA drives lipid metabolism, increasing TG via dihydroxyacetone phosphate (DHAP) upregulated as well and involved in glucose metabolism. DHAP, one of the breakdown products of fructose 1,6-bisphosphate, is also directly involved in energy storage, mediating TG synthesis. By contrast, in the resistant system LDHA inhibition only led to an increase in lipid markers of cell death. We also observed a reduction in amount of CE lipid family upon FASN inhibition, particularly drastic in tamoxifen-resistant condition whereas without treatment the basal levels remained high. These results are in line with other evidence in literature suggesting that interference on lipid metabolism alters structural elements, lipid synthesis, and degradation impairing tumorigenesis [45]. Specifically, a robust correlation has been demonstrated between CE-rich tumors and higher histologic grade, Ki-67 and tumor necrosis. This underscores that intratumoral CE accumulation is closely associated with the proliferation and aggressiveness of BC [46]. This approach provides a direct assessment of metabolic intracellular fluxes and their potential connections to crucial biological activities in BC revealing the metabolite and lipid context modification following FASN and LDHA inhibition.

Other studies associated metabolic phenotype to BC tumor aggressiveness. TNBC models were shown to be less efficient in aerobic respiration than ER + BC model and to be highly glycolytic [47–51]. About tamoxifen-resistant condition, in vitro studies report that chronic tamoxifen treatment indirectly initiated metabolic adaptations with decreased aerobic respiration and deregulated mitochondrial function by altering several complexes involved in aerobic respiration associated with decreased mitochondrial function [52–54].

In this study, we characterized the effect on proliferation and metabolic ability deeply investigating the metabolomic and lipidomic amount content upon the modulation of two enzymatic genes candidated for target metabolic therapy. FASN, identified as an oncogene, through its inhibition using omeprazole and TVB-2640 is the focal point of two clinical trials (NCT02595372 and NCT03179904). The concluded NCT02595372 trial demonstrated that the use of omeprazole in BC patients during chemotherapy led to a significant improvement in overall survival. While NCT03179904 is still in the recruitment phase and not yet completed, preliminary literature results have described a favorable tolerability and safety profile for TVB-2640 [55]. Currently, omeprazole, a FASN inhibitor, is FDA approved, chronically used, and well tolerated in clinical trials. About LDHA, due to its involvement in tumorigenesis and progression, LDHA inhibitors are good candidates as anticancer drugs. Although more experimental and clinical investigations are necessary to establish patient stratification. Target metabolic therapy should take in account metabolic vulnerabilities contextualized to the energy dependencies of specific tumor types and state of disease progression as crucial to drive therapeutic strategy.

In the last decades, metabolic reprogramming demonstrated a crucial role in the delopment of chemotherapy resistance. This is primarily because chemotherapy agents used in clinical settings induce compensatory metabolic reprogramming in cancer cells [56–59]. This study reveals the complex energy dynamics of BC, exploring the interplay between two crucial metabolic regulators, FASN and LDHA, and disease progression unveiling patterns linked to tumor aggressiveness and treatment response. Then, we characterized the metabolic modulation upon enzymatic inhibition showing the metabolic adaptability in tamoxifen-resistant condition. These results highlight BC heterogeneity and remark targeted metabolic therapy supporting approch for personalized medicine.

### Supplementary Information

Additional file 1.

Additional file 2.

Additional file 3.

Additional file 4.

Additional file 5.

Additional file 6.

Additional file 7.

## Data Availability

The data and materials of this study are available from the corresponding author upon request. For metabolomics and lipidomics data are deposited to zenodo ( https://zenodo.org/ ) DOI: https://doi.org/10.5281/zenodo.10370597 .

## Abbreviations

ATP: Adenosine triphosphate
BC: Breast cancer
CE: Cholesteryl esters
DG: Diacylglycerols
DEG: Differentially expressed genes
FASN: Fatty acid synthase
FAs: Fatty acids
HER2: Human epidermal growth factor receptor 2
LDHA: Lactate dehydrogenase A
Lum A: Luminal A
Lum B: Luminal B
MytoOCR: Mitochondrial oxygen consumption rate
NT: Normal tissue
OCR: Oxygen consumption rate
PR: Partial remission
PC: Phosphatidylcholines
PPP: Pentose phosphate pathway
PI: Phosphatidylinositol
PD: Progressing disease
PER: Proton efflux rate
PS: Phosphatidylserines
wTEAIT: With tumor event after initial treatment
woTEAIT: Without tumor event after initial treatment
