# Supplementary material for: Inverse FASN and LDHA correlation drives metabolic resistance in breast cancer
Source: J Transl Med. 2024 Jul 24;22:676. doi: 10.1186/s12967-024-05517-9 (PMC11267768; doi:10.1186/s12967-024-05517-9)
Supplement: Supplementary file 1 — Additional file 1. [file 12967_2024_5517_MOESM1_ESM.pdf]

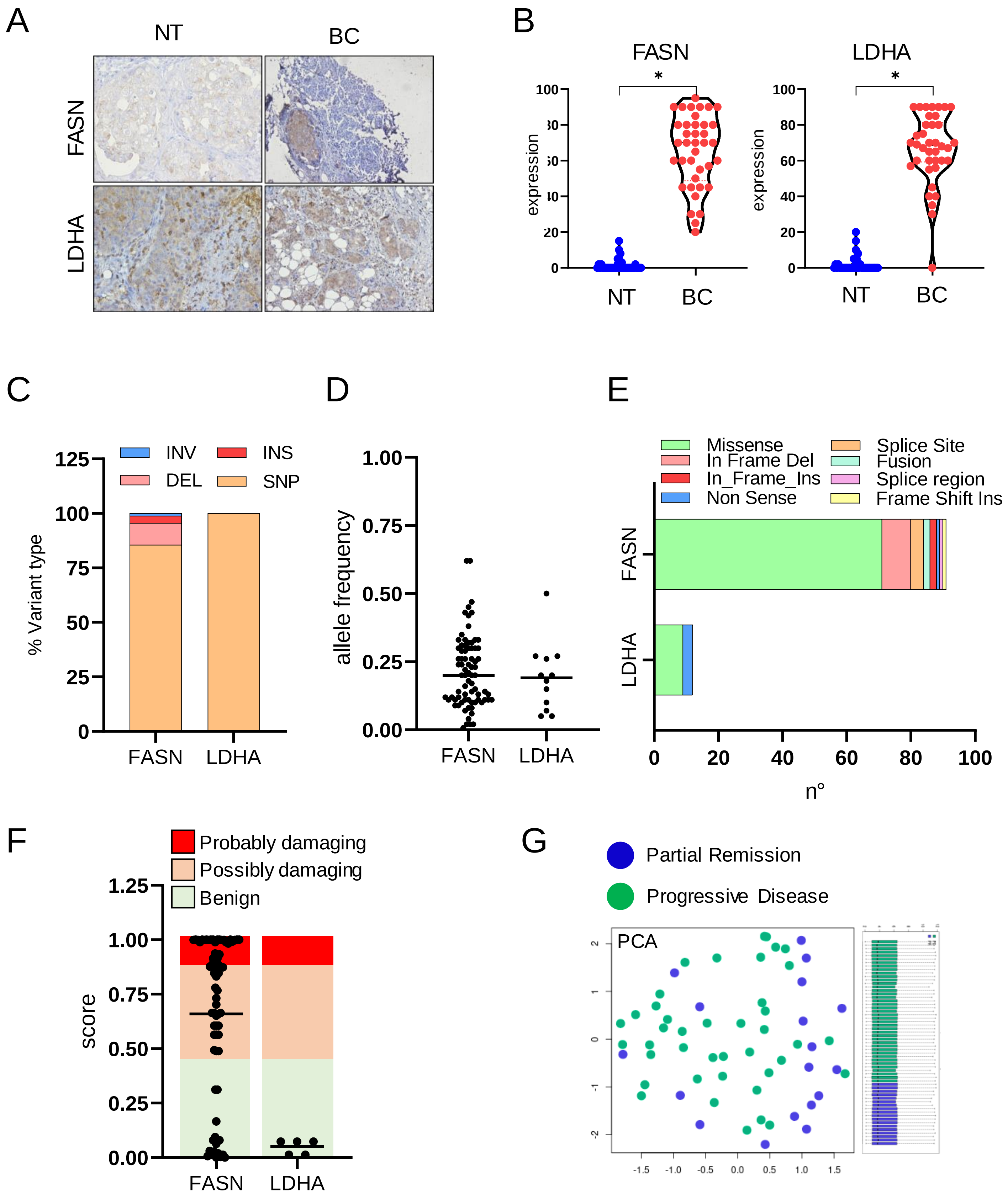

Supplementary Figure 1: **Mutational status of FASN and LDHA in BC patients. A**) Representative images of IHC data showing FASN and LDHA protein expression in BC and NT counterpart. **B)** Protein expression of FASN and LDHA in BC and NT counterpart IHC samples. Statistical significance showed as * pvalue <0.05. **C)** Variant type mutations in BC patients for FASN and LDHA genes reported in percentage and indicated as Inversion (blue), Insertion (red), Deletion (pink), and SNP (orange). **D**) Allele frequency of *FASN* and *LDHA* SNP mutations in BC patients. **E**) Number of mutations for FASN and LDHA in BC patients. Bars colored based on mutation type, as indicated in the legend. **F**) Score for FASN and LDHA missense mutations in BC patients. Predictive consequence is classified as probably damaging (red), possibly damaging (orange), and benign (green). **G)** Principal Component Analysis of BC database with partial remission (PR, in blue, n: 18) and progressing disease (PD, in green, n: 44) patients.

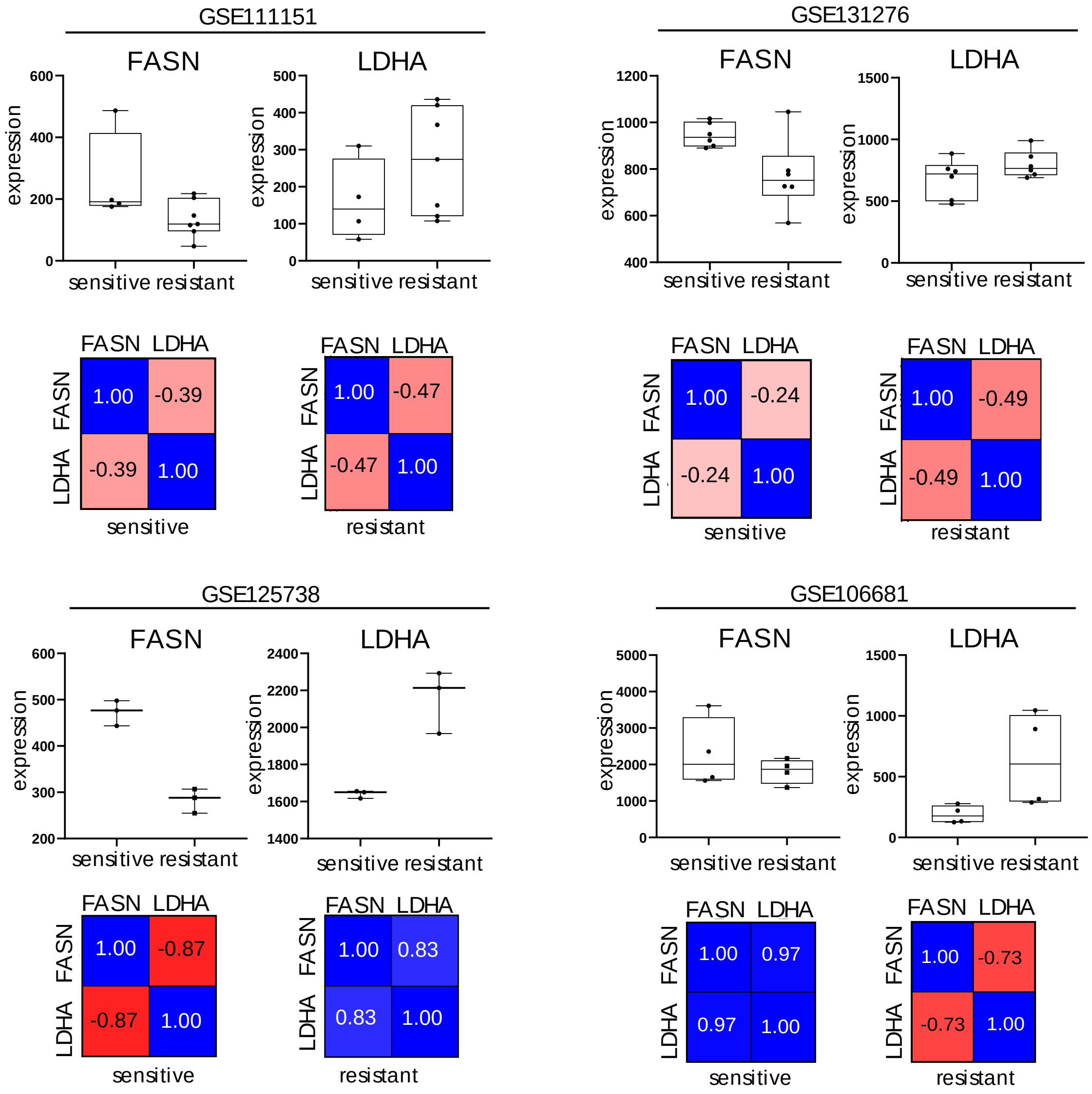

Supplementary Figure 2: FASN and LDHA correlation across sentisitive and resistant conditions. FASN and LDHA RNA-seq data in sensitive and resistant BC systems. Data reported as normalized expression. Pearson correlation for FASN and LDHA expression in sensitive and resistant BC systems reported as colorimetric scale blue (positive) and red (negative).

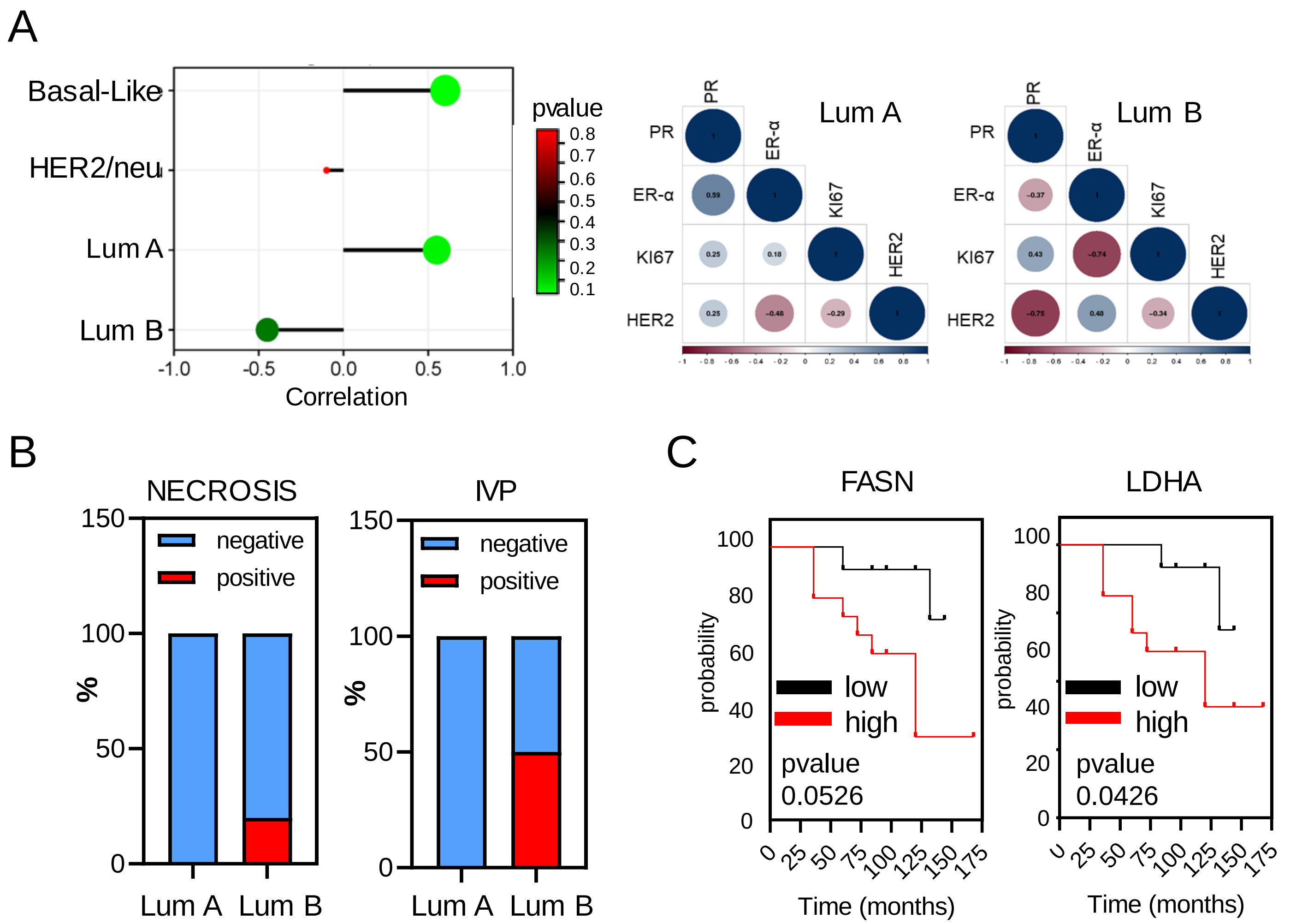

Supplementary Figure 3: FASN and LDHA protein expression in BC cohort and *in vitro* evaluation upon metabolic inhibition. A) Pearson correlation between ER-a and NR3C, human epidermal growth factor 2 (HER2) and Ki67 in the Luminal A (left) and Luminal B (right) BC subgroups reported as colorimetric scale blue (positive) and red (negative). **B)** Percentage of positivity for necrosis and IVP markers associated with the Lum A and Lum B groups. Bars colored as negative (blue) and posive (red) to marker expression. **C)** Kaplan–Meier curve showing survival probability in BC patients based on *FASN* and *LDHA* expression (black = low; red = high).

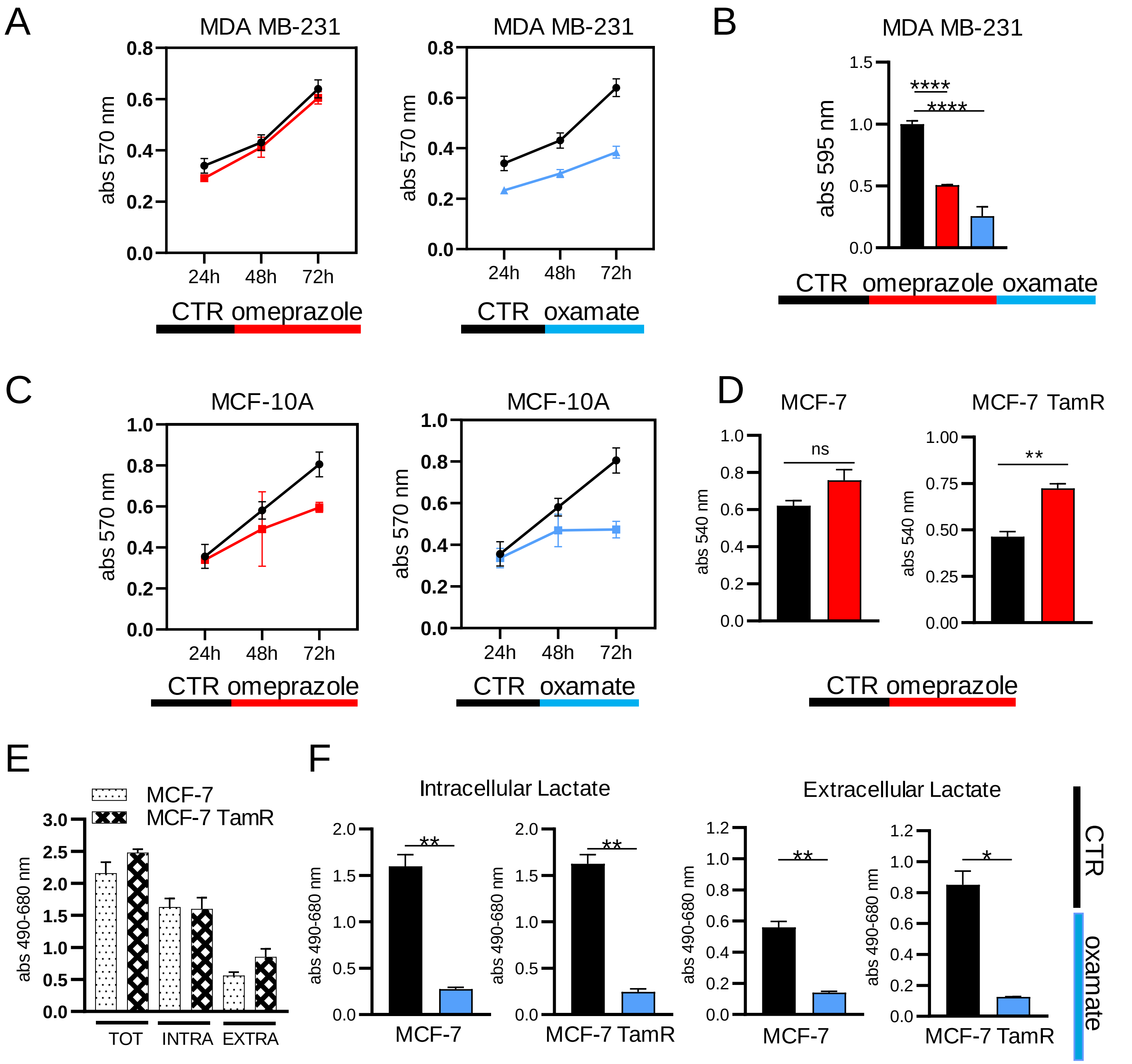

Supplementary Figure 4: A) Viability assay in MDA MB-231 cells upon FASN inhibitor omeprazole (red) and LDHA inhibitor oxamate (blue). **B)** Colony formation assay in MDA MB-231 after omeprazole (red) and oxamate treatment (blue). Values are mean ± standard deviation (SD) of biological replicates. Statistical significance showed as **** pvalue <0.0001. **C)** Viability assay in MCF-10A cells upon FASN inhibitor omeprazole (red) and LDHA inhibitor oxamate (blue). **D)** Red Oil assay in MCF-7 and MCF-7 TamR after omeprazole treatment. Histograms show absorbance values as mean ± standard deviation (SD) of biological replicates. Statistical significance showed as ** pvalue <0.01; ns, not significant. **E)** Lactate measurement in MCF-7 and MCF-7 TamR at basal level. Histograms show respectively total, intracellular, and extracelluar lactate. Absolute values are mean ± standard deviation (SD) of biological replicates. **F)** Intracellular, and extracelluar lactate in MCF-7 and MCF-7 TamR following oxamate. Histograms show absolute values for ctr (black) and oxamate (blue) treatment. Values are mean ± standard deviation (SD) of biological replicates. Statistical significance showed as * pvalue <0.05, ** pvalue <0.01.

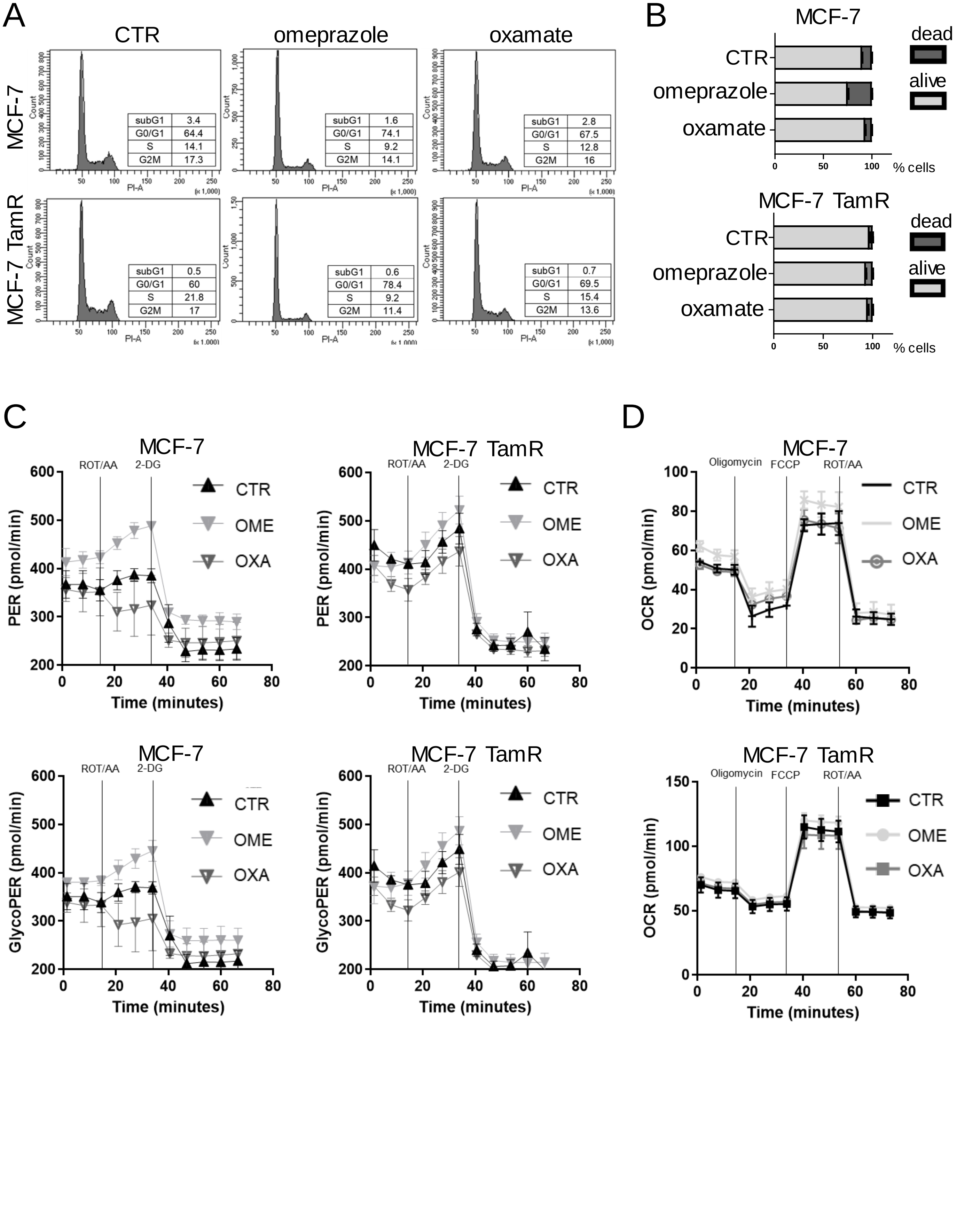

Supplementary Figure 5: Proliferative and energy traits under tamoxifen-resistance condition. **A)** Cell cycle analysis in MCF-7 and MCF-7 TamR after omeprazole and oxamate treatment. Graphs show the distribution of subG1, G0/G1, S, and G2M phases as percentage. **B)** Propidium iodide (PI) positivity in MCF-7 and MCF-7 TamR after omeprazole and oxamate treatment. Graphs of PI distribution shows live cells (light grey) and dead cells (dark grey). **C)** PER and GlycoPER measurement in MCF-7 (left side) and MCF-7 TamR (right side) after omeprazole and oxamate treatment. **D**) OCR measurement in MCF-7 (upper) and MCF-7 TamR (lower) after omeprazole and oxamate treatment.

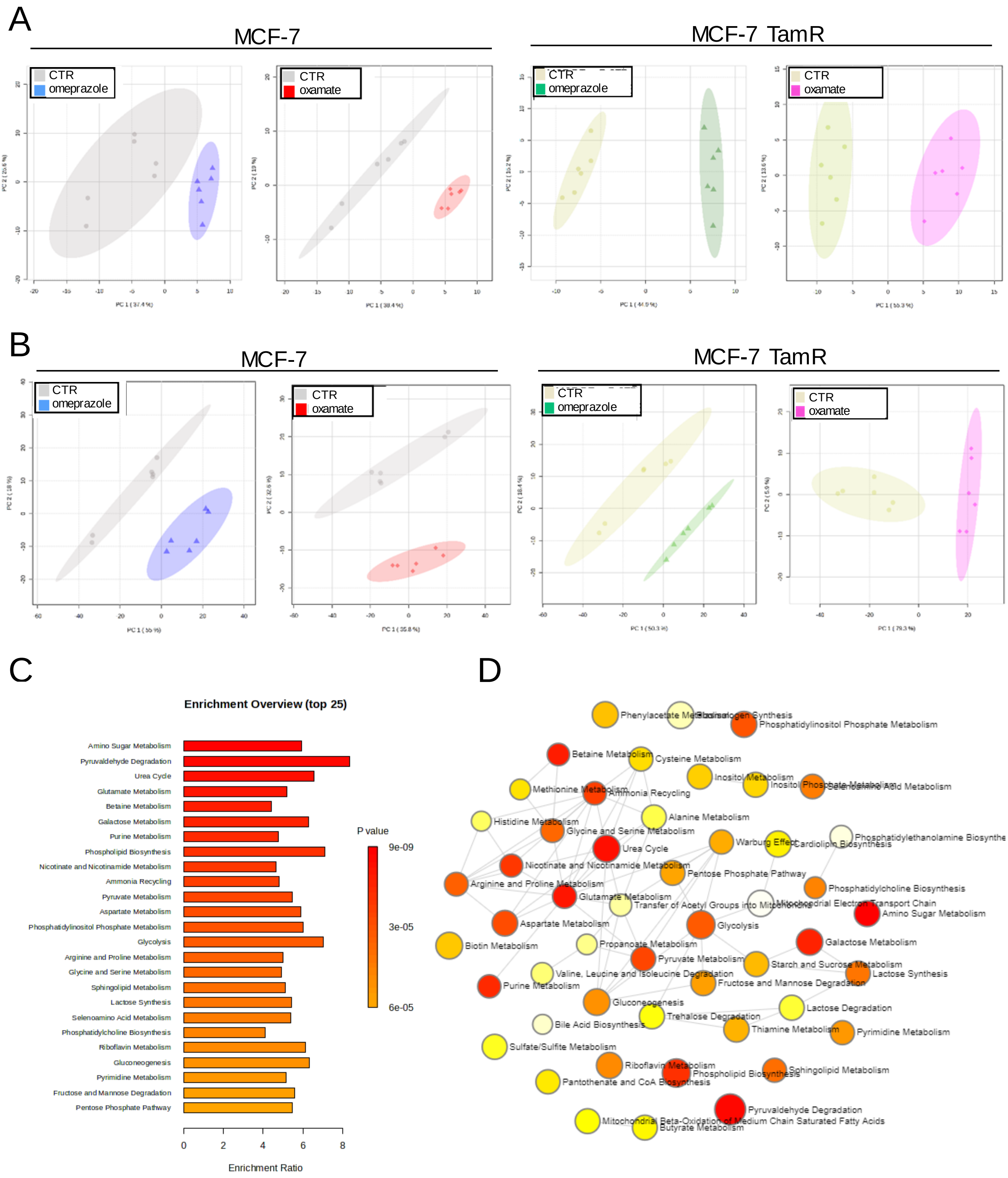

Supplementary Figure 6: Metabolomic and Lipidomic content reveal distint energy network. A-B) PCA plot showing respectively metabolomics and lipidomics datasets in MCF-7 CTR (grey) and MCF-7 TamR CTR (yellow) after omeprazole (blue/green) and oxamate (red/purple) treatment. **C)** Enrichment overview reporting the top 25 metabolic pathways from metabolomics dataset. Statistical significance reported as pvalue in a colorimetric scale (orange–red). **D)** Network analysis shows connection between pathways. Dimension knots refer to pathway size signature. Statistical significance reported as pvalue in a colorimetric scale (light yellow–red).

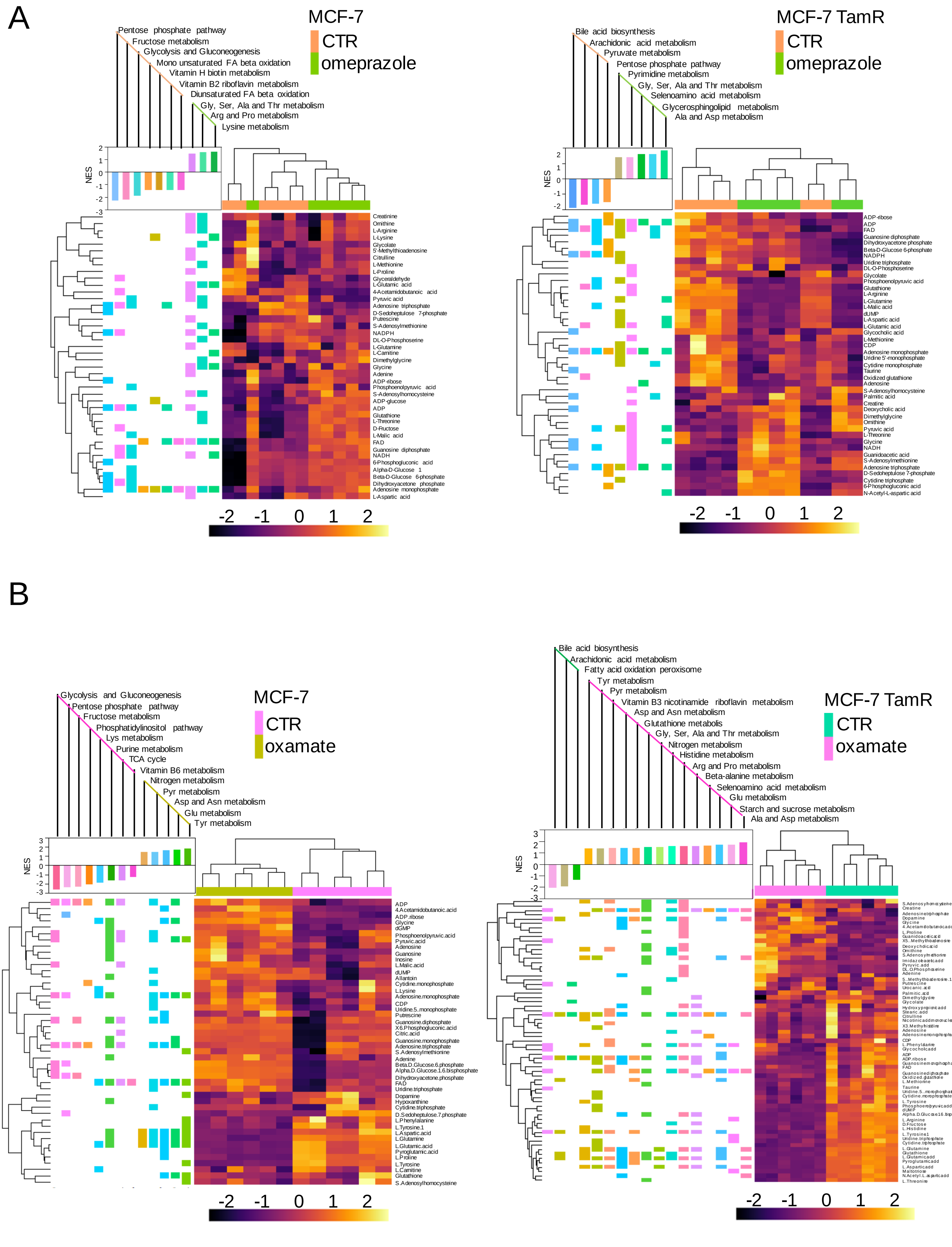

Supplementary Figure 7: Enrichement analysis in tamoxifen sensitive and resistant models. A-B) Heatmaps reporting Gene Set Enrichment Analysis as normalized enriched score (NES) for statistically significant differentially expressed metabolites in MCF-7 and MCF-7 TamR cells after omeprazole (upper panel) and oxamate (lower pannel) treatment.
